# Drought priming promotes phloem loading-driven sucrose export to alleviate the photosynthetic limitations under low nitrogen stress in wheat seedlings

**DOI:** 10.3389/fpls.2026.1798821

**Published:** 2026-04-14

**Authors:** Chengfeng Zhao, Liangyu Tao, Tongxi Bao, Xinyu Wang, Yujie Hu, Qiaomei Zheng, Zhongwei Tian, Tingbo Dai

**Affiliations:** 1 Key Laboratory of Crop Physiology Ecology and Production Management of Ministry of Agriculture, Nanjing Agricultural University, Nanjing, China; 2 Jinhua Academy, Zhejiang Chinese Medical University, Jinhua, Zhejiang, China

**Keywords:** drought priming, low nitrogen stress, photosynthesis, rubisco activation, sucrose export, wheat

## Abstract

Reducing nitrogen (N) application can improve nitrogen use efficiency (NUE) and mitigate environmental pollution. However, this reduction often adversely affects photosynthesis and leads to inhibition of wheat growth. Thus, exploring effective agronomic measures to improve wheat’s tolerance to low N is essential for balancing the conflict between N reduction and wheat growth. Drought priming has been proven to enhance plant stress tolerance, but its role and mechanisms in improving photosynthetic capacity under low N stress remain unclear. The effects of drought priming on wheat growth and photosynthesis were investigated under low N stress using hydroponic experiments with two wheat varieties, YM158 (low N-tolerant) and YM25 (low N-sensitive). The results showed that low N stress significantly inhibited biomass accumulation, whereas drought priming effectively alleviated this growth inhibition. Drought priming significantly increased the net photosynthetic rate (*P*_n_) of both cultivars compared to non-primed treatments under low N stress, which was primarily associated with enhanced Rubisco maximum carboxylation rate (*V*cmax) and activities of Rubisco and Rubisco activase (RCA). Furthermore, drought priming promoted triose phosphate (TP) utilization and upregulated the expression of sugar transporter genes, which reduced sucrose accumulation in leaves and consequently alleviated its feedback inhibition on photosynthesis. In summary, drought priming enhances phloem loading-driven sucrose transport, reduces leaf sucrose accumulation, and improves Rubisco activation, collectively alleviating photosynthetic feedback inhibition and sustaining stronger photosynthetic capacity under low N stress.

## 1 Introduction

The low nitrogen use efficiency (NUE) and elevated environmental risks associated with excessive nitrogen (N) application pose significant challenges in modern wheat production systems (Liu et al., 2020). One option for addressing this issue in production involves decreasing the application of basal N fertilizer (Gao et al., 2018). However, currently widely cultivated wheat cultivars have primarily been bred under conditions of abundant N supply, exhibiting heightened sensitivity to low N environments. Consequently, attempts to reduce N fertilizer usage have been hindered by the limited ability of crop plants to adapt to low N environments, often resulting in a significant decline in photosynthetic capacity and marked growth inhibition (Van Oosten et al., 2019).

Photosynthesis is a key limiting factor for plant dry matter accumulation and crop growth (Lv et al., 2025). This process converts CO_2_ into carbohydrates using light energy and drives the translocation of sucrose and other photoassimilates via the phloem from source to sink organs, providing essential carbon skeletons and energy for plant growth and development (Shah et al., 2017). Photosynthesis is driven by light energy and is also subject to feedback regulation by its own metabolic products (Paul and Pellny, 2003). N nutrition is a critical environmental factor influencing the export and utilization of photosynthetic products. Under N-deficient conditions, the demand for photoassimilates by nitrate reduction and other biosynthetic processes decreases, leading to the accumulation of carbohydrates such as sucrose in leaves, which subsequently triggers feedback inhibition of photosynthesis (Li et al., 2020). Carbohydrate accumulation typically downregulates the expression of photosynthesis-related genes, resulting in decreased photosynthetic rates (Stitt et al., 2010). Furthermore, sugar-mediated suppression of photosynthesis is also associated with a significant decline in the content, activation state, and activity of ribulose-1, 5-bisphosphate carboxylase/oxygenase (Rubisco) (Lobo et al., 2015). Although decreased Rubisco content and activity under insufficient N are well-established contributors to photosynthetic decline (Cetner et al., 2017), previous studies have predominantly attributed this reduction to impaired enzyme synthesis caused by N metabolic constraints, while overlooking the impact of sugar accumulation. Research has shown that excessive sucrose accumulation in the cytoplasm inhibits the transcription of Rubisco synthesis-related genes, thereby hindering its biosynthesis (McCormick et al., 2008). It has been reported that plants can enhance Rubisco activation state to compensate for protein loss under N-limitation, thereby sustaining photosynthetic capacity (Paul and Pellny, 2003; Gao et al., 2018). However, this compensatory mechanism is highly sensitive to leaf carbohydrate status. Accumulated sugars, particularly phosphorylated intermediates, can inhibit Rubisco activation (Amaral et al., 2024). Consequently, the inhibition of Rubisco activation by leaf sugar accumulation substantially compromises the efficacy of this compensatory mechanism under low N stress.

It is reported that high concentrations of phosphorylated sugars can bind to Rubisco and inactivate it (Amaral et al., 2024). During photosynthetic carbon metabolism, triose phosphate (TP), the precursor for sucrose synthesis, is exported from chloroplasts to the cytosol via the TP transporter located on the chloroplast envelope, coupled with the import of an equivalent amount of inorganic phosphate (Pi) into the chloroplasts, after which TP is converted into sucrose by sucrose phosphate synthase (SPS) in the cytoplasm with concomitant release of Pi (McClain and Sharkey, 2019). However, when sucrose accumulates to high levels in leaves, the suppression of SPS activity leads to impaired TP export, resulting in the retention of TP within chloroplasts, where it competes with RuBP for Rubisco catalytic sites and thereby inhibits its activity (Carmo-Silva et al., 2015). Rubisco activase (RCA) can restore Rubisco activity *in vivo* by removing tightly bound sugar phosphate inhibitors from its catalytic sites in an ATP-dependent manner (Amaral et al., 2024). Nevertheless, impaired TP export reduces the recycling of Pi back to chloroplasts, which in turn suppresses photophosphorylation and decreases ATP synthesis, ultimately leading to the inhibition of RCA activity (Zheng et al., 2023). Therefore, enhancing the capacity for TP export and its conversion to sucrose is crucial for maintaining Rubisco activity under low N stress.

Following its synthesis, sucrose must be transported to sink organs via the phloem (Nakad et al., 2022). Phloem loading is the first crucial step in sucrose transport to sinks, and its efficiency largely dictates the export capacity of photoassimilates from leaves, thereby influencing the strength of feedback inhibition (Li et al., 2022). Wheat primarily relies on the apoplastic pathway for sucrose loading (Aoki et al., 2004). The SUT and SWEET families of sugar transporters are key proteins in apoplastic phloem loading, with SWEETs responsible for effluxing sucrose into the apoplastic space and SUTs mediating sucrose uptake from the apoplast into companion cells and sieve elements (Liu et al., 2025). Studies have shown that environmental conditions such as temperature, water availability, and nutrient status can modulate phloem loading in leaves by regulating the expression of these sugar transporters (Li et al., 2018; Kaur et al., 2021). Under N-limiting conditions, enhancing preferential sugar translocation to roots to drive root growth and N uptake is a key adaptive strategy in plants, which is related to sugar export (Huang et al., 2022). A study in rice also demonstrated that low N stimulates the expression levels of SUTs and SWEETs, thereby enhancing phloem loading capacity in leaves and stems and improving carbon translocation to grains (Li et al., 2022). Therefore, enhancing sugar transporter-mediated phloem loading is likely of significant importance for improving low N tolerance.

The expression of sugar transporters is also regulated by plant water status (Kaur et al., 2021). Studies have shown that under water deficit conditions, the expression of *AtSWEET11*, *AtSWEET12*, and *AtSUC2* in Arabidopsis leaves is significantly upregulated to enhance carbon export from leaves to roots (Durand et al., 2016). Another study further revealed that drought signaling enhances the sucrose transport activity of *AtSWEET11* and *AtSWEET12* transporters through phosphorylation, thereby promoting root growth in Arabidopsis (Chen et al., 2022). Additionally, the expression of the *OsSUT2* gene in rice leaves is also upregulated under drought stress (Ibraheem et al., 2011). A systematic study analyzing the effects of multiple stresses, including drought, on the expression of SUTs in five dicot and four monocot species found that drought stress similarly affects SUTs expression in all monocot plants, specifically upregulating *SUT1* while downregulating *SUT4* (Xu et al., 2018). Collectively, these findings suggest that drought signaling promotes leaf sucrose loading and translocation by upregulating specific sugar transporters. Based on these findings, implementing appropriate drought priming strategies could be considered to strongly activate sugar export capacity in plant leaves when subjected to low N stress.

Drought priming, which involves pre-exposing plants to mild drought stress episodes to enhance their resistance to subsequent severe stress, has emerged as a promising strategy for improving the adaptive capacity of plants (Wang et al., 2014). This approach has been shown to significantly improve plant tolerance to subsequent abiotic stresses by inducing epigenetic modifications and modulating primary metabolic processes (D’Urso and Brickner, 2014; Wang et al., 2023). Multiple studies in wheat have confirmed that drought priming positively regulates physiological responses to drought and high-temperature stress, resulting in improved photosynthetic performance and dry matter accumulation in primed plants under stress conditions (Mendanha et al., 2020; Kumar et al., 2023; Sun et al., 2025). However, most existing research has explained the alleviation of stress-induced photosynthetic inhibition primarily through enhanced antioxidant defense and reduced stomatal limitations, with few studies addressing the improvement in photosynthetic capacity from the perspective of sugar export and carbon partitioning regulation. It remains unclear whether and how drought priming facilitates sucrose export from leaves to mitigate photosynthetic feedback inhibition under N-deficiency.

We hypothesize that drought priming can alleviate sugar-mediated feedback inhibition of photosynthesis by enhancing Rubisco activation and phloem loading-driven sucrose export, ultimately improving photosynthetic performance and growth of wheat seedlings under low N stress. In this study, we measured changes in photosynthetic capacity, photosynthetic enzyme activities, sucrose synthesis and metabolism, as well as phloem loading capacity in wheat leaves to elucidate the mechanisms by which drought priming improves photosynthesis under low N stress. This research aims to explore viable approaches and their theoretical foundations for stable wheat production under low N conditions.This study aims to explore viable approaches and theoretical basis for nitrogen reduction and efficient cultivation of wheat.

## 2 Materials and methods

### 2.1 Plant material and experimental design

The hydroponic experiment was carried out at Nanjing Agricultural University, Nanjing, China, in 2023. Two wheat (*Triticum aestivum* L.) cultivars, Yangmai158 (YM158, low N-tolerant) and Yangmai25 (YM25, low N-sensitive), were used in the experiment. Seeds of uniform size were disinfected with 15% (v/v) H_2_O_2_ for 10 min, washed with distilled water 6 times, and then germinated at 20°C in plastic containers lined with two layers of wet sterilized gauze in darkness until the root length reached approximately 1 cm. Thereafter, well-germinated seeds were transferred to sterilized specialized seedling pots and grown with distilled water. After full expansion of the first leaf, seedlings were moved into 35 L plastic containers filled with half-strength modified Hoagland nutrient solution (Jiang et al., 2017) for 3 days. They were then supplied with full-strength modified Hoagland nutrient solution until the three-leaf stage was reached.

Drought priming treatment was applied at the three-leaf stage. Half of the seedlings of each cultivar were subjected to 10% PEG-6000 (φ = –0.56 MPa) for 4 days, which induced a mild reduction in leaf relative water content (RWC, **Supplementary Figure 1**), followed by a three-day recovery period in normal Hoagland solution. Subsequently, both the control and drought-primed seedlings were randomly divided into two subgroups: sufficient N (5 mM N) application controls and low N (0.25 mM N) stressed plants (detailed nutrient composition provided in **Supplementary Table 1**). The low N treatment lasted for 10 days. That is, four treatments were established: no drought priming plants supplied with sufficient N (Control), drought priming plants supplied with sufficient N (Priming), no drought priming plants supplied with low N (Non-priming + low N), drought priming plants supplied with low N (Priming + low N). Each treatment contained three replicates, each replication consisted of 3 containers, with 60 plants per container. The containers were arranged randomly and re-randomized weekly to reduce greenhouse location effects. During the test period, the containers were placed in a greenhouse under a 16 h light/8 h dark photoperiod at 18/8.5 °C, 400 μmol m^-2^ s^-1^ light intensity, and 60% humidity. The nutrient solutions with different treatments were changed every three to four days, aerated continuously throughout the experiments, and adjusted with H_2_SO_4_ or NaOH daily to maintain the pH between 5.5 and 6.0.

### 2.2 Plant sampling and morphological analysis

After 5 and 10 days of low N treatment (DAT), 90 uniform wheat seedlings from each treatment were randomly harvested and washed with distilled water. Half of the seedlings were divided into three groups (15 seedlings per group) as three independent biological replicates. To ensure that each biological replicate represented the population mean of the entire replicate, the 15 seedlings in each group were pooled from all three containers within that replicate (i.e., five seedlings per container). Plants within containers were pooled prior to analysis to avoid pseudoreplication. Young leaves (the fifth leaf on the main stem, grown and fully expanded during low N stress) and old leaves (the third leaf on the main stem, fully expanded functional leaves before drought priming treatment) were collected from these seedlings. The leaf samples were flash-frozen in liquid nitrogen for 12 h and then stored at -80°C for subsequent chemical analysis. The selection of these two leaf positions was based on the physiological response differences observed among different leaf positions under low N stress (Chai et al., 2024), which may affect the drought priming effect. Comparative analysis between young and old leaves enables a systematic investigation into the overall adaptation mechanisms of the plant.

Similarly, the remaining half of the seedlings were also divided into three groups, and the plants were separated into leaves, stems, and roots. Leaf area was measured using an area meter (Li-3000, Li-Cor Inc., Lincoln, NE, USA). The separated organs of each plant were first dried at 105°C for 30 min and then dried to a constant weight at 75°C. The dry matter was then used for measuring dry weight and for the quantification of sucrose content.

### 2.3 Relative water content measurement

Leaf relative water content (RWC) was determined following the method described by Lv et al. (2024). On day 4 of drought priming treatment, the third fully expanded leaf from the main stem was collected, and its fresh weight (FW) was recorded immediately. The leaf was placed in a sealed bag filled with deionized water and stored in darkness at 4°C for 24 h. After removal, surface water was gently blotted off with absorbent paper, and the turgid weight (TW) was recorded. The leaf was then oven-dried at 75°C for 48 h to constant weight, and the dry weight (DW) was recorded. RWC was calculated as: RWC (%) = [(FW – DW)/(TW – DW)] × 100.

### 2.4 Leaf gas exchange measurement

Leaf gas exchange measurements were performed at 1, 3, 5, 7 and 10 DAT, using a portable photosynthesis system LI-COR 6400 (Li-Cor Inc., Lincoln, USA) with a 6-cm^2^ leaf area chamber to measure the net photosynthetic rate (*P*_n_), stomatal conductance (*g*_s_), and intercellular CO_2_ concentration (*C*_i_) on the old and young leaves. The measurements were conducted from 09:30 to 11:30 with photosynthetic photon flux density (PPFD) of 1200 μmol m^−2^ s^−1^, temperature of 25 °C, flow rate of 500 μmol s^−1^, and the ambient CO_2_ concentration of 400 μmol mol^−1^. The relative humidity was controlled between 55%-65%, and the vapor pressure deficit (VPD) was maintained at 1.1 kPa.

The *A*/*C*_i_ curve was measured on the old and young leaves at 5 and 10 DAT, using the same apparatus, PPFD, temperature, flow rate, and relative humidity as mentioned above. The key difference was that leaf gas exchange parameters were recorded across a gradient of CO_2_ concentrations (400, 300, 200, 100, 50, 400, 600, 800, 1000, 1200, 1500, 1800 μmol · mol^−1^), and the data were recorded for about 2–3 min per setting. The *A*/*C*_i_ curves were analyzed according to Sharkey et al. (2007) based on the model developed by Farquhar et al. (1980), to estimate the Rubisco maximum carboxylation rate (*V*cmax) and triose phosphate utilization rate (*V_TPU_*).

### 2.5 Rubisco content, activity and activation state determination

Rubisco content was determined following the methods of Makino et al. (1986); Zheng et al. (2023), with minor modifications. Leaf samples (0.3 g) were ground under freezing conditions, mixed thoroughly with 5 mL of extraction buffer containing 50 mM Tris-HCl (pH 7.6), 1 mM MgCl_2_, 12.5% (v/v) glycerol, 5 mM β-mercaptoethanol, and 1 mM EDTA; then centrifuged at 10, 000 × g for 15 min at 4°C. Next, 1.6 mL of the crude extract was mixed with 0.4 mL of 5× loading buffer, boiled for 5 min, and centrifuged at 2, 000 × g for 15 min. A 10 μL aliquot of the supernatant was separated by 4-12% SDS-PAGE (120 V, 1 h). After electrophoresis, the gel was stained with 0.25% (w/v) Coomassie Brilliant Blue R-250 for 1.5 h, followed by destaining with a solution of 25% ethanol and 8% acetic acid until the background became transparent. The bands corresponding to the large subunit (56 kDa) and small subunit (14 kDa) of Rubisco were excised, dissolved in 1 mL of formamide, and decolorized overnight at 50 °C with shaking. Absorbance of the eluates was measured at 595 nm using a spectrophotometer, with protein concentrations determined from a bovine serum albumin (BSA) standard curve.

The initial activity of Rubisco was assayed according to the method of Gao et al. (2018) with modifications. Fresh leaf tissue (0.1 g) was ground to a fine powder in liquid nitrogen and homogenized in 1 mL of ice-cold extraction buffer containing 50 mM HEPES (pH 7.5), 1 mM EDTA, 10 mM MgCl_2_, 12.5% (v/v) glycerol, 1% (w/v) polyvinylpolypyrrolidone (PVPP), and 10 mM β-mercaptoethanol, then centrifuged at 15, 000 × g for 20 min at 4°C to obtain the crude enzyme extract. 100 μL of supernatant was combined with 700 μL of reaction buffer [50 mM HEPES (pH 8.0), 1 mM EDTA, 10 mM MgCl_2_, 20 mM NaHCO_3_, 5 mM dithiothreitol (DTT), 5 mM ATP-Na_2_, 0.2 mM NADH-Na_2_, 10 U/mL creatine phosphokinase, 10 U/mL 3-phosphoglycerate kinase, 10 U/mL glyceraldehyde-3-phosphate dehydrogenase, and 5 mM phosphocreatine] in a quartz cuvette. The reaction was initiated by adding 200 μL of 3 mM ribulose-1, 5-bisphosphate (RuBP), followed by immediate mixing. The decrease in absorbance at 340 nm was monitored at 25°C for 60 s with measurements recorded at 10-s intervals using a UV-visible spectrophotometer. Rubisco activity was calculated based on the reaction rate, with one unit of enzyme activity (U) defined as the amount of enzyme required to oxidize 1 nmol of NADH per minute per gram of fresh leaf tissue at 25°C.

The Rubisco activation state was calculated as the ratio of initial Rubisco activity to total Rubisco activity. Total Rubisco activity was determined according to the method of Gao et al. (2018). Briefly, 100 μL of Rubisco enzyme extract was incubated with an equal volume of activation solution (containing 40 mM MgCl_2_ and 20 mM NaHCO_3_) at 25°C for 10 min to achieve enzyme activation. The subsequent assay steps and calculation formula were identical to those described previously for the initial Rubisco activity measurement.

### 2.6 Rubisco activase activity determination

Rubisco activase (RCA) activity was assayed using established protocols from Carmo-Silva and Salvucci (2011); Gao et al. (2018). Briefly, 50 μL of Rubisco extract was rapidly mixed with 50 μL of Rubisco inactivation buffer (containing 10 mM RuBP and 5% (v/v) polyethylene glycol 3350), followed by incubation at 4 °C for 5 minutes. Subsequently, 50 μL aliquots of this mixture were separately incubated with an equal volume of reactivation solution (containing 90 mM Tris-HCl pH 7.5, 40 mM MgCl_2_, 20 mM NaHCO_3_, with or without 10 mM ATP-Na_2_, 10 mM phosphocreatine, and 200 U mL^-1^ phosphocreatine kinase) at 25 °C for 5 minutes. Rubisco activity was then monitored at 30 s intervals for 5 min after reaction initiation. The fraction of catalytically restored Rubisco active sites was determined by comparison with total Rubisco activity. RCA activity was quantified based on the increase in the proportion of active Rubisco sites between 1.5 and 3 min, as measured in reactions lacking ATP.

### 2.7 Inorganic phosphorus concentration determination

The content of inorganic phosphorus (Pi) was determined according to the method described by Zheng et al. (2023). Approximately 0.1 g of frozen leaf tissue was homogenized in 1 mL of glacial acetic acid (v/v, 1:1), followed by centrifugation at 12, 000 × g and 4 °C for 15 min. Then, 0.1 mL of the supernatant was mixed with 2 mL of FeSO_4_-(NH_4_)_2_MoO_4_ reagent and allowed to react for 1 minute at room temperature. After thorough mixing, the absorbance was measured at 660 nm. The Pi content was calculated based on a standard curve of phosphorus.

### 2.8 Sucrose phosphate synthase activity assays

The activity of sucrose phosphate synthase (SPS) in the leaves was assayed as described by Li et al. (2022), with minor modifications. Frozen leaf samples (0.1 g) were homogenized in 1 mL 50 mM HEPES-NaOH buffer (pH 7.5) and then centrifuged at 15, 000 × g for 15 min at 4 °C to obtain the crude enzyme extract. A 30 µL enzyme extract was combined with 100 µL of reaction buffer (containing 50 mM HEPES-NaOH pH 7.5, 15 mM UDP-glucose, 15 mM fructose-6-phosphate, and 10 mM MgCl_2_) and incubated for 15 min at 30 °C. The reaction was terminated with 100 µL of 2 M NaOH. To remove interfering fructose-6-P, the mixture was heated in a boiling water bath for 10 min. Sucrose was quantified colorimetrically by adding 3.5 mL of 30% (w/v) HCl and 1 mL of 0.1% (w/v) resorcinol, incubating at 80 °C for 10 min, and measuring the absorbance at 480 nm.

### 2.9 Sucrose content determination

Approximately 0.05 g of dry leaf tissue was extracted by 5 mL of 80% (v/v) ethanol three times at 80 °C for 10 min, and then centrifuged at 4, 000 × g for 10 min at 25 °C. This extraction procedure was repeated three times. After each centrifugation step, the supernatants were pooled and brought to a final volume of 15 mL using 80% ethanol. The combined supernatant was then decolorized by adding 0.05 g of activated carbon, followed by centrifugation at 4, 000 × g for 10 min at 25 °C. The resulting supernatant was collected and used for the determination of sucrose content. The leaf sucrose content was measured using the resorcinol method and estimated on the basis of the absorbance at a wavelength of 480 nm according to Hu et al. (2019).

### 2.10 Expression analysis of sucrose transport-related genes

Total RNA was extracted from the old and young leaves using the Plant RNA Extraction Kit (Vazyme). The RNA samples were then subjected to cDNA synthesis using the PrimeScript RT reagent kit with gDNA Eraser (Vazyme) following the manufacturer’s instructions. Gene expression analysis was performed via real-time qPCR following Zheng et al. (2024), with *TaADP-RF* serving as the reference gene. The stability of *TaADP-RF* under diverse experimental conditions has been previously validated in wheat (Paolacci et al., 2009), and its reliable expression has been confirmed in numerous subsequent studies (Zheng et al., 2024; Sun et al., 2025). Primer sequences for *TaADP-RF* (reference gene) (Paolacci et al., 2009), *TaSUT1* (Gao et al., 2024), *TaSUT2* (Navabpour and Jalali, 2019), and *TaSWEET11* (Wang et al., 2025) have been reported previously. All primer sequences are listed in **Supplementary Table 1**. There were three biological replicates and two technical replicates for each gene. The relative expression of these genes was analyzed using the 2^-ΔΔCt^ method described by Livak and Schmittgen (2001).

### 2.11 Statistical analysis

Statistical analyses were performed using IBM SPSS Statistics version 25.0 for the Windows system (SPSS Inc., Chicago, IL, USA). Prior to statistical analysis, all data were tested for normality and homogeneity of variances. Differences in *P*_n_, *g*_s_, and *C*_i_ among treatments were assessed by one-way ANOVA. For all other parameters, a three-way ANOVA was applied to test the effects of cultivar, priming, N-level, and their interactions (cultivar × priming, cultivar × N-level, priming × N-level, and cultivar × priming × N-level). Where significant effects were found (*P* < 0.05), means were separated using Duncan’s multiple range test. All data are presented as mean ± standard deviation (SD). The corresponding F-values from the three-way ANOVA are presented in **Supplementary Tables 3**-**S8**. Figures were generated using Origin 2025 (OriginLab Corp., Northampton, MA, USA).

## 3 Results

### 3.1 Wheat seedlings growth

**Figure 1:**
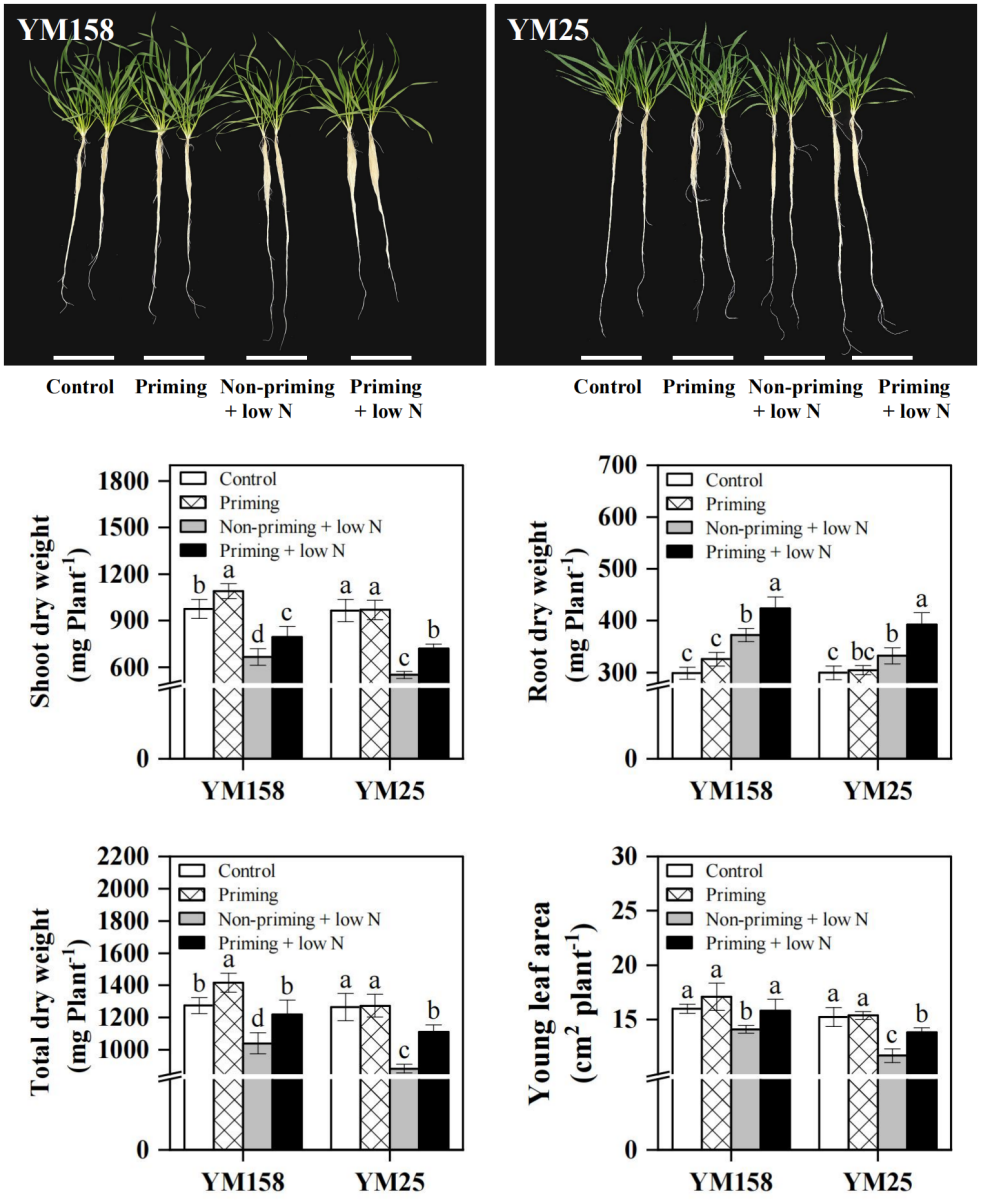
Effect of drought priming on the plant growth status of wheat seedlings under low nitrogen stress. Values (means ± SD, n = 3) bearing different letters differ significantly at *P* < 0.05 according to the Duncan’s Multiple Range test. Control, no drought priming plants supplied with sufficient N; Priming, drought priming plants supplied with sufficient N; Non-priming + low N, no drought priming plants supplied with low N; Priming + low N, drought priming plants supplied with low N.

Following 10 days of low N stress, both wheat cultivars exhibited significant (*P* < 0.05) growth inhibition, as evidenced by reduced shoot dry weight, total dry weight, and young leaf area (**Figure 1**). However, drought priming substantially alleviated these inhibitory effects. Specifically, under low N stress, primed plants of the tolerant cultivar YM158 showed increases of 19.2% in shoot dry weight, 17.2% in total dry weight, and 12.0% in young leaf area compared to non-primed controls. Notably, these beneficial effects were more pronounced in the sensitive cultivar YM25, which exhibited greater improvements of 30.5%, 25.8%, and 18.4% in the respective traits, suggesting that the priming conferred a stronger advantage to the sensitive variety. In contrast to its impact on shoots, low N stress itself significantly increased root dry weight, and this increase was further enhanced by drought priming in both cultivars. Interestingly, under sufficient N conditions, drought priming also significantly promoted shoot dry biomass accumulation in YM158.

### 3.2 Photosynthetic parameters

**Figure 2:**
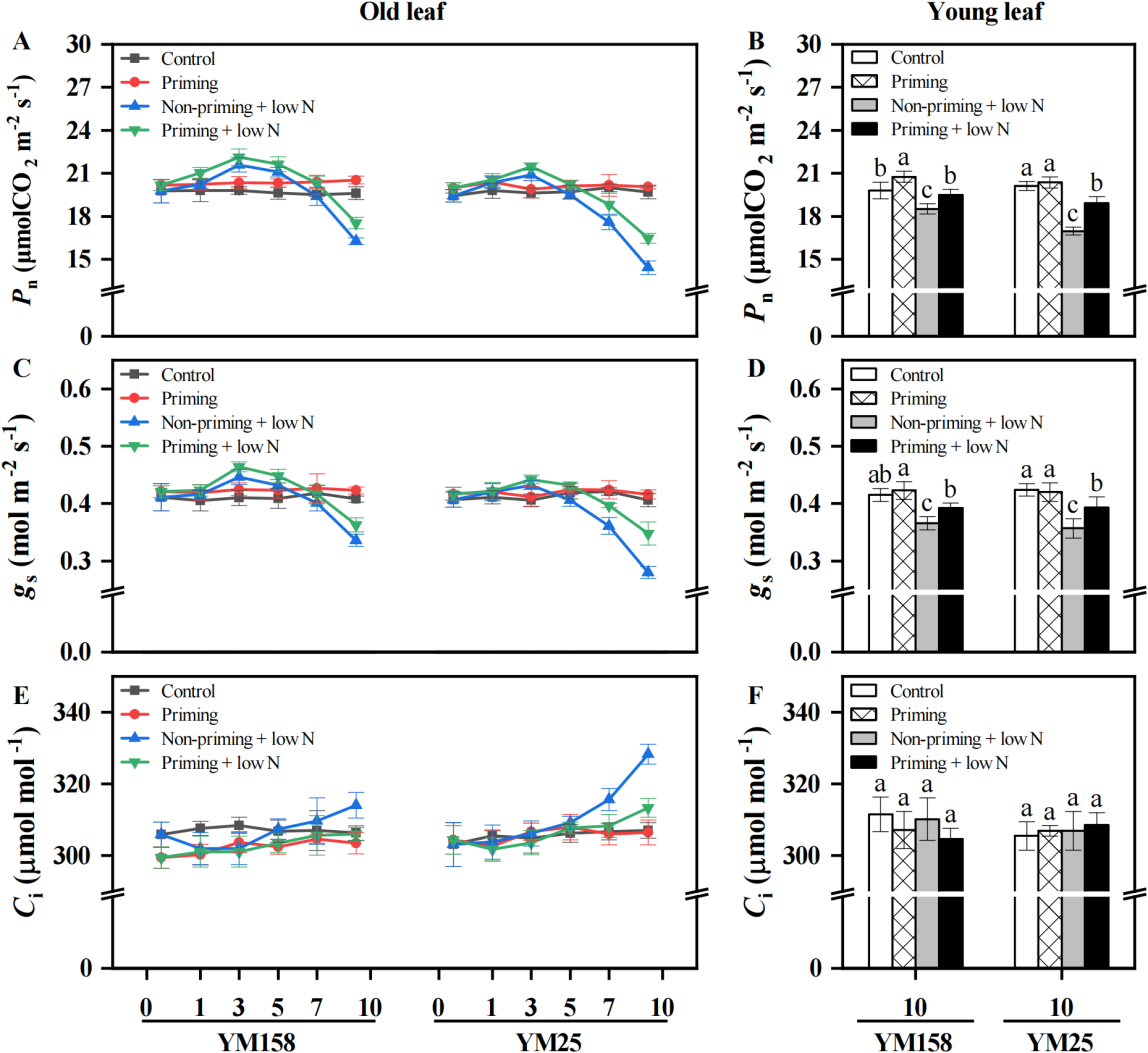
Effect of drought priming on gas exchange characteristics of wheat seedlings leaves under low nitrogen stress. Values (means ± SD, n = 3) bearing different letters differ significantly at *P* < 0.05 according to the Duncan’s Multiple Range test. Control, no drought priming plants supplied with sufficient N; Priming, drought priming plants supplied with sufficient N; Non-priming + low N, no drought priming plants supplied with low N; Priming + low N, drought priming plants supplied with low N.

As a key physiological indicator characterizing biomass accumulation, *P*_n_ directly reflects the plant’s carbon assimilation capacity. Following exposure to low N stress, the *P*_n_ and *g*_s_ of old leaves in both cultivars showed a significant initial increase (*P* < 0.05) followed by a significant decline, but the sensitive cultivar exhibited an earlier decline compared to the tolerant cultivar (**Figure 2**). Specifically, YM158 exhibited a 17% reduction in *P*_n_ at 10 DAT relative to the control, while YM25 showed a significant decline as early as 7 DAT, with reductions of 12% (at 7 DAT) and 26.6% (at 10 DAT). For young leaves, the *P*_n_ of both varieties decreased significantly at 10 DAT, by 6.5% in YM158 and 15.6% in YM25 relative to the control. In contrast, drought priming treatment markedly alleviated the decline in *P*_n_ in wheat leaves under low N stress, when compared to untreated plants. Changes in *g*_s_ were co-ordinated with *P*_n_. In addition, *C*_i_ in older leaves of both species remained at a comparable level to that of control plants during the early stage of low N stress but showed a significant increase (*P* < 0.05) during the later stage of low N stress, especially in non-primed plants (**Figure 2**). However, in young leaves, no significant differences in *C*_i_ were observed between the low N stress group and the control group in either cultivar, irrespective of drought priming treatment. Similarly, the low N-sensitive cultivar YM25 received a greater benefit from drought priming in terms of gas exchange parameter improvement than the tolerant cultivar YM158. Under sufficient N conditions, *P*_n_ of young leaves in YM158 seedlings subjected to drought priming was better than that of untreated seedlings, while there was no significant difference in YM25.

### 3.3 Rubisco activity and *V*cmax

**Figure 3:**
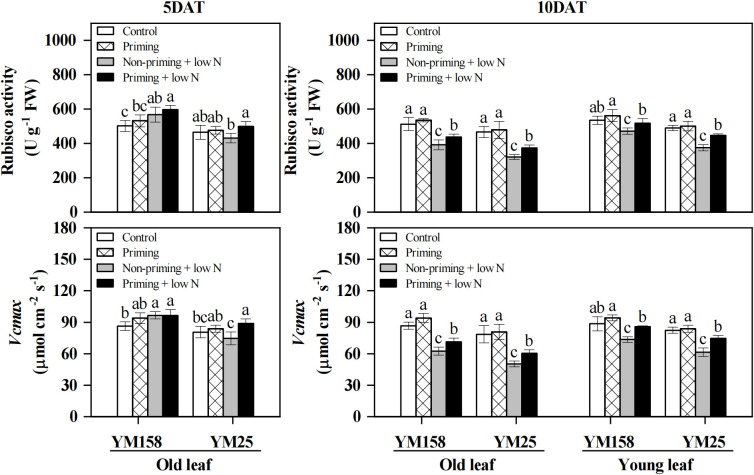
Effect of drought priming on Rubisco activity and maximum carboxylation rate (*V*cmax) of wheat seedlings under low nitrogen stress. Values (means ± SD, n = 3) bearing different letters differ significantly at *P* < 0.05 according to the Duncan’s Multiple Range test. Control, no drought priming plants supplied with sufficient N; Priming, drought priming plants supplied with sufficient N; Non-priming + low N, no drought priming plants supplied with low N; Priming + low N, drought priming plants supplied with low N.

When exposed to low N treatment for 5 days, a significant increase (*P* < 0.05) in Rubisco activity and *V*cmax was observed in non-primed YM158, while it showed a slight but statistically insignificant decrease in non-primed YM25 (**Figure 3**). Upon continued stress until day 10, both old and new leaves of the two cultivars demonstrated significantly lower Rubisco activity and *V*cmax relative to controls. Drought priming significantly alleviated the decline in Rubisco activity and *V*cmax induced by low N stress. Compared with low N treatment alone, drought-primed old and young leaves of YM158 exhibited 11.8% and 14.9% enhancements in Rubisco activity, and 14.2% and 16.8% increases in *V*cmax, respectively, at 10 DAT. For YM25, drought priming exerted significant effects as early as 5 days under low N stress. Relative to non-primed plants, primed old leaves showed Rubisco activity increases of 15.7% and 16.3%, and *V*cmax increases of 15.9% and 20.5% at the two time points, respectively. By 10 DAT, primed young leaves also exhibited significant enhancements, with Rubisco activity reaching 446.08 U g^-1^ FW compared to 374.81 U g^-1^ FW in non-primed plants (a 19.0% increase), and *V*cmax reaching 74.57 μmol cm^-2^ s^-1^ compared to 61.54 μmol cm^-2^ s^-1^ (a 21.2% increase).

### 3.4 Rubisco content and activation

**Figure 4:**
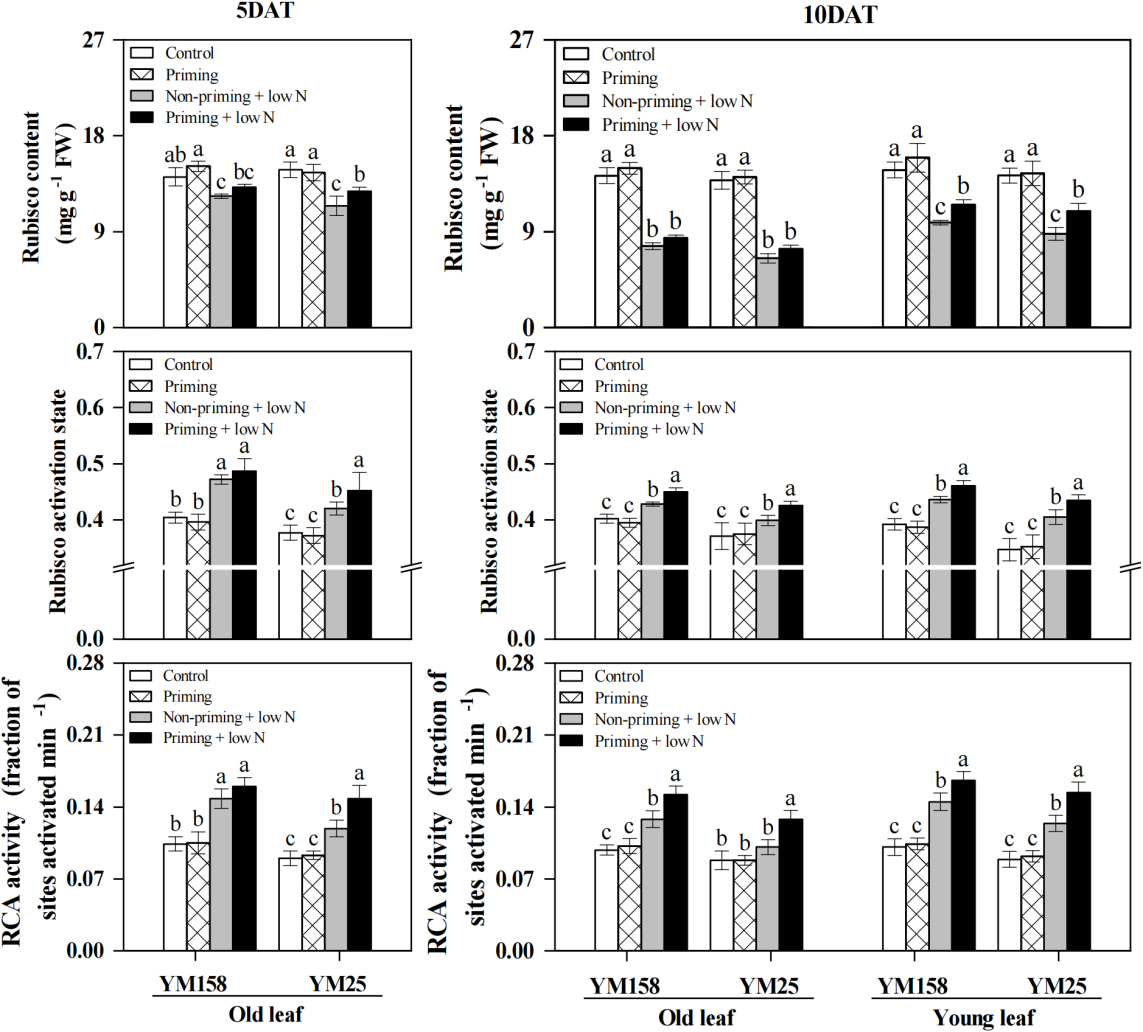
Effect of drought priming on Rubisco content, Rubisco activation state and Rubisco activase (RCA) activity of wheat seedlings under low nitrogen stress. Values (means ± SD, n = 3) bearing different letters differ significantly at *P* < 0.05 according to the Duncan’s Multiple Range test. Control, no drought priming plants supplied with sufficient N; Priming, drought priming plants supplied with sufficient N; Non-priming + low N, no drought priming plants supplied with low N; Priming + low N, drought priming plants supplied with low N.

Low N treatment significantly reduced (*P* < 0.05) Rubisco content in both old and young leaves of the two cultivars, while drought priming alleviated this decline in young leaves, but had no significant effect on the old leaves of the two cultivars (**Figure 4**). In contrast, low N stress significantly enhanced (*P* < 0.05) the activation state of Rubisco and the activity of RCA in both cultivars (except in the old leaves of YM25 at 10 DAT) (**Figure 4**). Drought priming further enhanced Rubisco activation state and RCA activity under low N stress. Compared to non-primed plants, YM158 plants subjected to drought priming displayed significant enhancements in Rubisco activation state and RCA activity at 10 DAT, in both old and young leaves. For the sensitive cultivar YM25, the enhancement occurred earlier and was more pronounced. Significant increases of 7.5% in activation state and 24.0% in RCA activity were observed as early as 5 DAT. By 10 DAT, the increases reached 6.4% and 26.3% in old leaves, and 7.2% and 24.5% in young leaves, respectively.

### 3.5 *V_TPU_* and leaf Pi concentration

**Figure 5:**
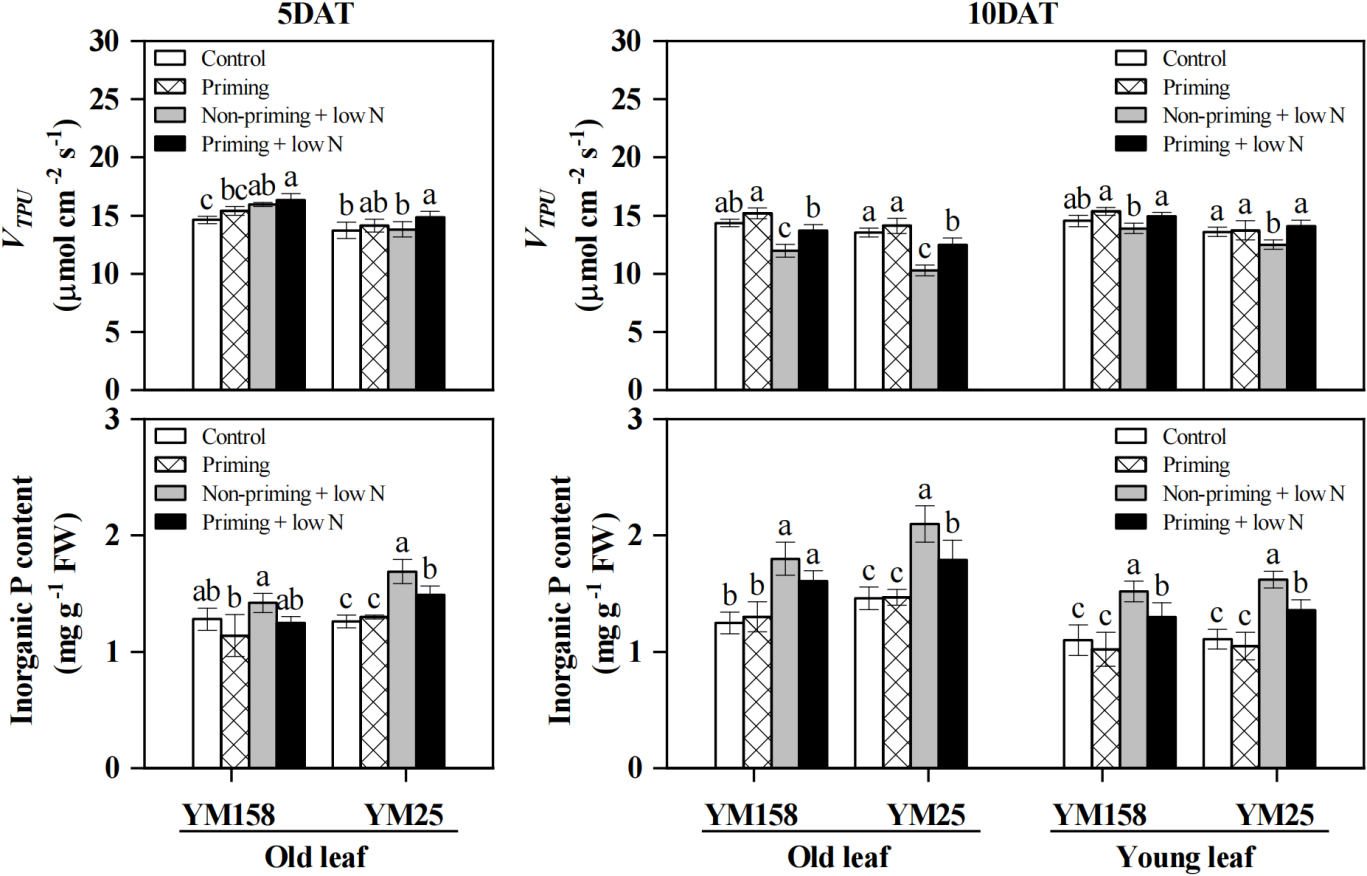
Effect of drought priming on triose phosphate utilization rate (*V_TPU_*) and leaf inorganic phosphorus (Pi) concentration of wheat seedlings leaves under low nitrogen stress. Values (means ± SD, n = 3) bearing different letters differ significantly at *P* < 0.05 according to the Duncan’s Multiple Range test. Control, no drought priming plants supplied with sufficient N; Priming, drought priming plants supplied with sufficient N; Non-priming + low N, no drought priming plants supplied with low N; Priming + low N, drought priming plants supplied with low N.

Under sufficient N supply conditions, no significant differences in leaf *V_TPU_* were observed between the two cultivars (**Figure 5**). After 5 DAT, the *V_TPU_* of YM158 was significantly higher (*P* < 0.05) than that of the sufficient N supply control, regardless of drought priming, with no significant difference between the primed and non-primed groups (**Figure 5**). In contrast, YM25 showed a significant increase in *V_TPU_* only when subjected to drought priming (with a 12.1% increase relative to the control), while the low N treatment alone did not differ significantly from the control. At 10 DAT, low N stress significantly suppressed *V_TPU_* in the old leaves of both cultivars and the young leaves of YM25, whereas the *V_TPU_* in YM158 young leaves was maintained at a level comparable to the control. Relative to low N treatment alone, drought priming at 10 DAT markedly enhanced *V_TPU_* in both old and new leaves of the two cultivars, with increases of 14.2% and 7.5% in YM158, and 21.1% and 12.6% in YM25, respectively.

Following low N treatment for 5 days, the Pi content in YM158 leaves showed no significant change, while a substantial accumulation (*P* < 0.05) was observed in YM25 leaves (**Figure 5**). By 10 DAT, significant Pi accumulation occurred in both old and young leaves of the two cultivars, with a more pronounced accumulation in the old leaves. Drought priming effectively alleviated the low N stress-induced Pi accumulation. Specifically, at 5 DAT, low N stress increased leaf Pi content in YM25 by 34.7% compared to the control, whereas in drought-primed plants, the increase was only 18.5%. At 10 DAT, compared to low N stress alone, drought priming reduced Pi content by 10.4% and 14.6% in the old and young leaves of YM158, respectively, and by 15.0% and 15.7% in those of YM25.

### 3.6 SPS activity and sucrose content

**Figure 6:**
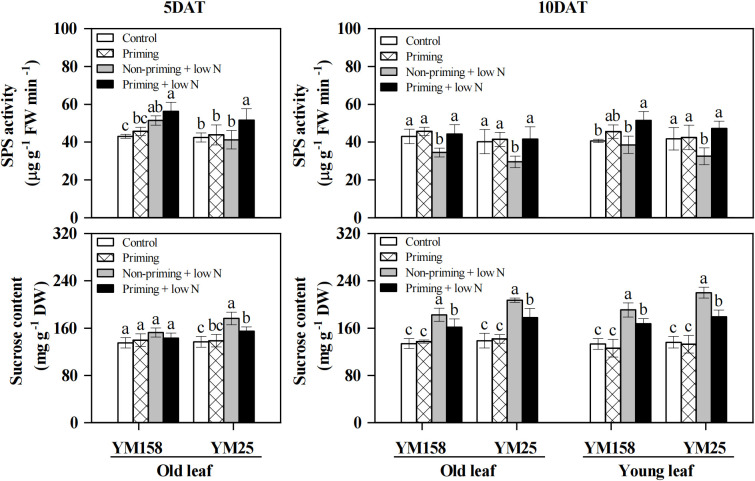
Effect of drought priming on sucrose phosphate synthase (SPS) activity and sucrose content in wheat seedlings under low nitrogen stress. Values (means ± SD, n = 3) bearing different letters differ significantly at *P* < 0.05 according to the Duncan’s Multiple Range test. Control, no drought priming plants supplied with sufficient N; Priming, drought priming plants supplied with sufficient N; Non-priming + low N, no drought priming plants supplied with low N; Priming + low N, drought priming plants supplied with low N.

Low N stress significantly increased (*P* < 0.05) SPS activity in YM158 at 5 DAT, but led to a general decrease by 10 DAT in both cultivars and leaf types (**Figure 6**). The only exception was the young leaves of YM158, which showed no significant difference from the control. In contrast, drought priming markedly enhanced SPS activity in both old and young leaves under low N stress (except in YM158 at 5 DAT). Measurement of leaf sucrose content revealed that low N stress significantly increased (*P* < 0.05) sucrose content in both old and young leaves of the two cultivars, with YM25 exhibiting an earlier and more pronounced accumulation (**Figure 6**). Drought priming significantly alleviated the low N-induced sucrose accumulation in the leaves. At 10 DAT, priming reduced sucrose content in YM158 old and young leaves by 11.5% and 12.2%, respectively; in YM25, priming decreased old leaf sucrose content by 12.1% at 5 DAT and 14.1% at 10 DAT, and young leaf sucrose content by 18.5% at 10 DAT.

### 3.7 Expression of sugar transporter genes

**Figure 7:**
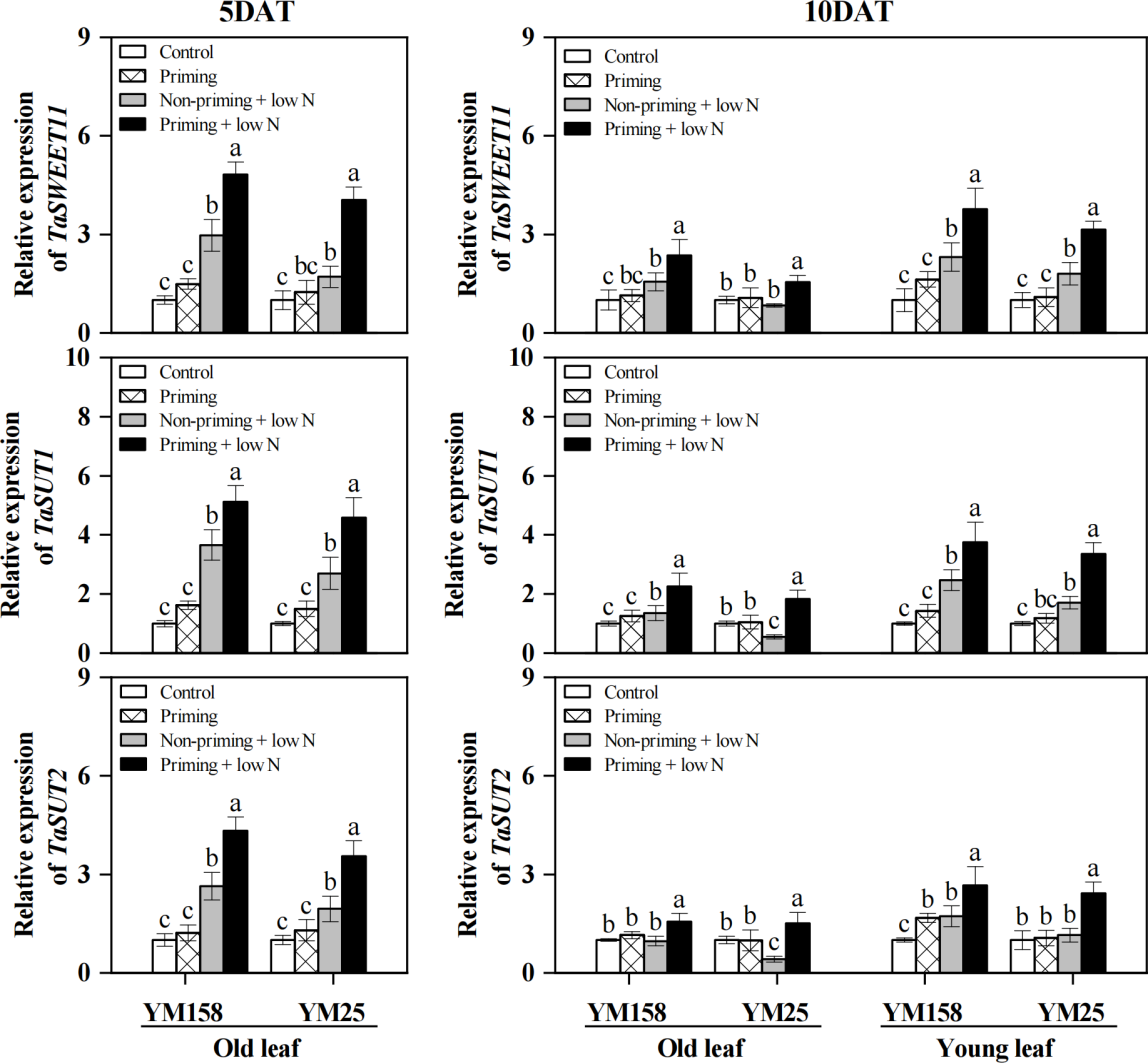
Effect of drought priming on the relative expression of sugar transporter genes of wheat seedlings under low nitrogen stress. Values (means ± SD, n = 3) bearing different letters differ significantly at *P* < 0.05 according to the Duncan’s Multiple Range test. Control, no drought priming plants supplied with sufficient N; Priming, drought priming plants supplied with sufficient N; Non-priming + low N, no drought priming plants supplied with low N; Priming + low N, drought priming plants supplied with low N.

After 5 days of low N stress, the transcript levels of sugar transporter genes *TaSWEET11*, *TaSUT1*, and *TaSUT2* were significantly upregulated (*P* < 0.05) in both cultivars, and drought priming further enhanced this upregulation (**Figure 7**). Following 10 days of low N treatment, the expression levels of *TaSWEET11*, *TaSUT1*, and *TaSUT2* gradually declined in leaves of both cultivars. Among them, the expression of *TaSWEET11* and *TaSUT1* remained significantly higher than the control in both old and young leaves of YM158, while in YM25, only young leaves maintained elevated expression, with expression in old leaves either significantly lower than or comparable to the control. *TaSUT2* expression remained higher than the control only in young leaves of YM158; it was similar to the control in old leaves of YM158 and young leaves of YM25, but significantly downregulated in old leaves of YM25. In contrast, at 10 DAT, the expression levels of *TaSWEET11*, *TaSUT1*, and *TaSUT2* in both old and young leaves of drought-primed plants were significantly higher than those in the non-primed group across both cultivars.

## 4 Discussion

Plant growth and development are highly dependent on N nutrient availability, and N-deficiency seriously inhibits the growth of crops (Liu et al., 2020). In this study, wheat seedlings under low N stress exhibited significant growth inhibition, manifesting as reduced shoot dry weight and young leaf area (**Figure 1**). The capacity of crops to maintain better growth under abiotic stress is regarded as a crucial stress tolerance trait. Drought priming has been demonstrated to enhance crop stress tolerance, with numerous studies confirming its positive effects on plant growth under abiotic stress conditions (Li et al., 2015; Abid et al., 2016; Lv et al., 2024). Similarly to previous results, our results showed that drought priming pretreatment enabled wheat seedlings to maintain higher shoot and root dry weight, as well as larger young leaf area under low N stress, significantly alleviating the inhibition of N-deficiency on plant growth (**Figure 1**). The improved growth of wheat was attributed to the mitigation and delay in the decline of *P*_n_ under low N stress by drought priming (**Figure 2**). Under low N stress, the decrease in *P*_n_ was accompanied by a decline in *g*_s_, yet *C*_i_ remained at control levels or even increased (**Figure 2**). Previous studies have suggested that the photosynthetic decline under nitrogen deficiency is primarily limited by biochemical factors rather than stomatal closure (Gao et al., 2018). In this study, the significant reduction in Rubisco content and activity under low N conditions provides direct evidence supporting this conclusion (**Figures 3**, **4**).

It was reported that N-deficiency-induced suppression of *P*_n_ initially results from impaired Rubisco carboxylation capacity (Cetner et al., 2017; Gao et al., 2018). In agreement with those results, in this experiment, wheat plants exhibited temporal dynamics in *V*cmax, showing stability or increase during early stress, and then decreased significantly with the duration of stress (**Figure 3**). The *V*cmax reflects Rubisco’s catalytic performance *in vivo*, being dependent on both the enzyme’s concentration and its activation status (Long and Bernacchi, 2003). The activation state of Rubisco is regulated by RCA, which activates catalytic activity by removing inhibitors from the active site of Rubisco, enhancing RCA activity is considered an important mechanism for adaptation to low N environments (Gao et al., 2018; Pasch et al., 2025). The observed increase or maintenance in *V*cmax could potentially be attributed to enhanced activation state of Rubisco, whereby plants upregulate RCA activity to compensate for the reduced Rubisco content under N-deficiency, thereby preserving net carbon assimilation capacity (Gao et al., 2018). This compensatory mechanism was further substantiated by our observation that wheat maintained or even enhanced Rubisco activity despite significant reductions in Rubisco content during the early stress phase (**Figure 4**). However, with continuing N-limitation, negative feedback caused by factors such as sugar accumulation may lead to the failure of Rubisco activation (as demonstrated by the old leaves of YM25), which in turn triggers large-scale mobilization of the photosynthetic apparatus (Paul and Pellny, 2003).

The impact of drought priming on Rubisco carboxylation capacity is debated. Some studies have shown that only severe drought stress caused a significant decrease in Rubisco activity, while mild drought intensity does not induce detectable changes in either Rubisco activity or protein abundance (Pelloux et al., 2001; Bota et al., 2004), suggesting that the intensity of drought priming may not influence Rubisco activity or content. However, other researchers have reported that drought-primed plants can enhance photosynthetic carbon assimilation under stress conditions by either increasing Rubisco content (Abid et al., 2016), upregulating the abundance of Rubisco small subunits (RbcS) and RCA (Wang et al., 2014). Similarly, transcriptomic studies in drought-treated grapevines revealed that one of the earliest responses to water deficit is an increase in the transcript abundance of RCA, which is considered a compensatory mechanism for the decrease in *g*_s_ (Cramer et al., 2007). Consistent with the latter perspective, our study demonstrated that drought priming significantly increased Rubisco content, Rubisco activation state, and RCA activity in young wheat leaves under low N stress (**Figure 4**). Notably, although drought priming had a limited effect on Rubisco content in old leaves compared to non-primed plants, it significantly enhanced Rubisco activation state and RCA activity, leading to increased overall Rubisco activity (**Figures 3**, **4**). These results indicate that drought priming improves Rubisco carboxylation efficiency primarily by enhancing Rubisco activation, thereby sustaining photosynthetic performance in wheat under N-deficient conditions.

Rubisco activation is critically dependent on ATP supply, which is sustained by Pi recycling through TP utilization (McClain and Sharkey, 2019; Zheng et al., 2023). When TP utilization is constrained, Pi becomes limited within chloroplasts, impairing photophosphorylation and ATP synthesis, while retained TP directly competes with RuBP for Rubisco catalytic sites, further suppressing carboxylation (Carmo-Silva et al., 2015; Fabre et al., 2019). In this study, low N stress significantly reduced *V_TPU_* in wheat leaves, whereas drought priming effectively alleviated this inhibition under nitrogen deficiency (**Figure 5**), suggesting that priming enhances TP export and thus supports Rubisco activation. Interestingly, total leaf Pi content increased under low N stress, but was significantly reduced by drought priming (**Figure 5**). Similar observations have been reported in maize and Arabidopsis, where N-deficiency leads to Pi accumulation in leaves due to reduced growth-induced Pi utilization and enhanced Pi uptake (Schlüter et al., 2012; Torres-Rodríguez et al., 2021). However, this apparent increase in total Pi does not contradict the notion of chloroplastic Pi-deficiency, as phosphorus partitioning among subcellular compartments differentially affects metabolic activity in each region (Schlüter et al., 2012). The reduced leaf Pi concentration observed in primed plants likely indicates more efficient Pi utilization, a trait closely associated with improved growth performance.

TP utilization is closely linked to sucrose synthesis, which is primarily regulated by SPS (Lobo et al., 2015). A robust sucrose synthesis capacity not only promotes TP export and phloem loading but also accelerates Pi recycling and supports Rubisco carboxylation (Fabre et al., 2019). In this study, SPS activity in non-primed plants declined significantly during the later stage of low N stress (**Figure 6**). It has been reported that excessive accumulation of carbohydrates such as sucrose in leaves inhibits SPS activity, impeding TP transport and reducing *P*_n_ (Zhang et al., 2024). N-deficiency has been shown to suppress plant growth, thereby reducing carbohydrate consumption and leading to substantial sucrose accumulation in leaves (Paul and Pellny, 2003; Zhang et al., 2024). Consistent with these findings, sucrose content in both young and old leaves of wheat under low N stress increased significantly in this study (**Figure 6**), explaining the observed decline in SPS activity. In contrast, drought priming alleviated sucrose accumulation and enhanced SPS activity under low N stress (**Figure 6**), enabling plants to maintain higher sucrose synthesis capacity and TP export efficiency, thereby mitigating photosynthetic feedback inhibition. These coordinated responses ultimately support the sustained enhancements in *P*_n_ and Rubisco activity observed in primed plants.

The lower level of sucrose accumulation observed under drought priming was likely attributed to an enhanced capacity for long-distance sucrose transport. Sugar transporters provide the key driving force for the long-distance movement of sucrose from source to sink organs (Chen et al., 2022). Studies have shown that drought and osmotic stress can induce the upregulation of multiple SWEET and SUT genes, thereby mobilizing greater sugar transport to sink organs such as the root system (Durand et al., 2016; Kaur et al., 2021). In this study, drought priming significantly elevated the expression levels of *TaSUT1, TaSUT2*, and *TaSWEET11* in leaves under low N stress (**Figure 7**), suggesting an enhanced capacity for phloem sucrose loading and transport under N-deficiency. This interpretation is supported by indirect evidence from sink organ responses. Primed plants exhibited significantly higher root dry matter accumulation and greater new leaf area under low N stress compared to non-primed plants (**Figure 1**). These increases in sink organ growth indicate enhanced sink demand and suggest that more photoassimilates were successfully transported from source leaves to sink tissues. Sucrose production and transport are primarily driven by sink demand, a strong sink enhances phloem export of sugars and facilitates Calvin cycle processes by accelerating Pi recycling and RuBP regeneration (Fabre et al., 2019). The promoted growth resulting from drought priming under low N stress underscores the critical role of a strong sink in driving a robust cycle of sugar transport and photosynthetic metabolism, which in turn reinforces growth and its maintenance. Nevertheless, we acknowledge that direct transport measurements would provide more conclusive evidence. Future studies employing ^14^C/^11^C pulse-labeling or phloem sap analysis are needed to definitively establish the link between transporter gene expression and actual carbon partitioning under drought-primed conditions.

Furthermore, in this study, we observed that drought priming significantly enhanced the growth of YM158 under sufficient N levels, whereas under low N conditions, its improving effect was more pronounced in YM25. This discrepancy suggests a strong overlap between drought and N stress signaling pathways (Plett et al., 2020). Previous studies have indicated that winter wheat with higher NUE often exhibits stronger drought tolerance under soil water deficit (Zhou et al., 2022). We speculate that for YM158, the 5 mM N level may exceed its optimal requirement, creating mild nitrogen excess stress, and the stress resistance mechanisms activated by drought priming partially alleviated this stress. The more pronounced priming effect in the N-sensitive YM25 under low N conditions can be attributed to its differential baseline physiological status. Our data show that YM25 accumulated more leaf sucrose and had lower sugar transporter expression than YM158, indicating a stronger susceptibility to end-product feedback inhibition. This resulted in a greater initial growth restriction, but also left more “room for improvement” via drought priming. By enhancing phloem loading and sucrose export, priming alleviated this inhibition more substantially in YM25, restoring its photosynthetic capacity to a greater extent. This highlights the cultivar-dependent nature of priming efficacy, which closely interacts with the plant’s intrinsic metabolic status under stress.

## 5 Conclusion

In summary, our results demonstrate that drought priming effectively alleviates low N-induced growth inhibition, enhances photosynthetic performance, and consequently improves low N tolerance in wheat. Drought priming improved the activation state of Rubisco by enhancing RCA activity, thereby increasing photosynthetic carboxylation efficiency under low N stress. Further analysis revealed that drought priming increases the utilization of TP and promotes the export of TP from chloroplasts. Concurrently, it upregulated sugar transporter expression to promote sucrose phloem loading. These coordinated actions reduced leaf sucrose accumulation, ultimately alleviating the sugar-mediated feedback inhibition of photosynthesis. Our findings extend the physiological understanding of drought priming in plant stress adaptation, and provide a feasible strategy for N reduction and efficient cultivation of wheat. However, the present study was conducted only under hydroponic conditions and focused solely on the seedling stage. Therefore, whether the drought priming effect during early growth can persist into the grain filling stage and benefit yield formation remains to be confirmed in field experiments, which will provide a practical strategy for sustainable wheat production.

## Data Availability

The original contributions presented in the study are included in the article/**Supplementary Material**. Further inquiries can be directed to the corresponding author/s.
