# Supplementary material for: Drought priming promotes phloem loading-driven sucrose export to alleviate the photosynthetic limitations under low nitrogen stress in wheat seedlings
Source: Front Plant Sci. 2026 Apr 14;17:1798821. doi: 10.3389/fpls.2026.1798821 (PMC13121060; doi:10.3389/fpls.2026.1798821)
Supplement: Supplementary file 1 [file DataSheet1.docx]

**
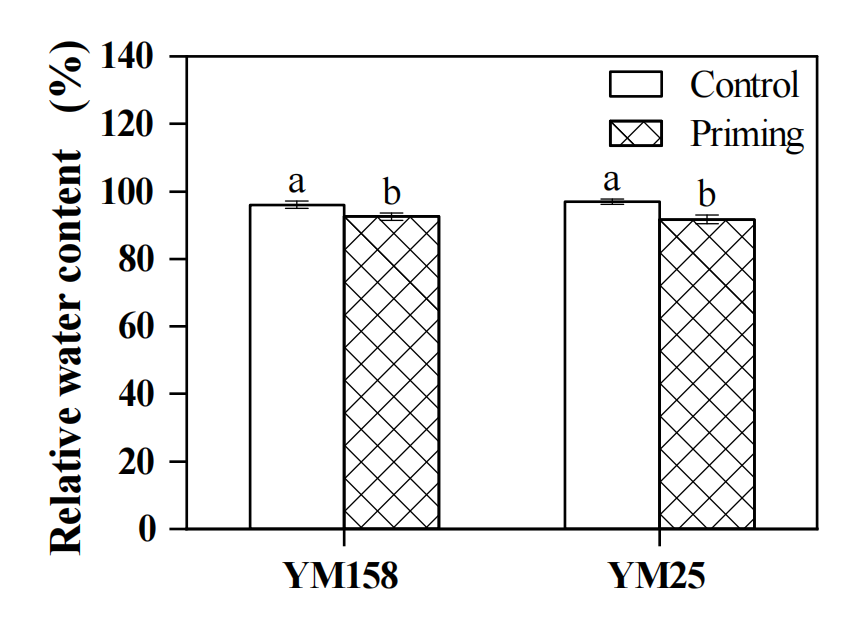
**

**Fig. S1** Changes in the relative water content of wheat seedling leaves after 4 days of drought priming treatment. Values (means ± SD, n = 3) bearing different letters differ significantly at *P* < 0.05 according to the Duncan’s Multiple Range test. Control, no drought priming plants supplied with sufficient N; Priming, drought priming plants supplied with sufficient N.

**Table S1** The formulations composition of nutrient solution in this study

| Composition | Sufficient nitrogen | Low nitrogen |
| --- | --- | --- |
| Ca(NO_3_)_2_•4H_2_O | 1.5 mM | 0.05 mM |
| KNO_3_ | 2 mM | 0.15 mM |
| K_2_SO_4_ | 0 mM | 0.925 mM |
| CaCl_2_ | 0 mM | 0.4 mM |
| CaSO_4_ | 0 mM | 1.05 mM |
| KH_2_PO_4_ | 1 mM | 1 mM |
| MgSO_4_.7H_2_O | 1 mM | 1 mM |
| NaCl | 0.5 mM | 0.5 mM |
| Fe-EDTA | 10 μM | 10 μM |
| H_3_BO_3_ | 2.35 μM | 2.35 μM |
| MnSO_4_·H_2_O | 0.55 μM | 0.55 μM |
| ZnSO_4_·7H_2_O | 0.0385 μM | 0.0385 μM |
| CuSO_4_·5H_2_O | 0.0165 μM | 0.0165 μM |
| H_2_MoO_4_ | 0.0065 μM | 0.0065 μM |

**Table S2** Primers used in qRT-PCR analysis

| Genes | Sequence |
| --- | --- |
| *TaADPRF*-Q-F | GCTCTCCAACAACATTGCCAAC |
| *TaADPRF*-Q-R | GCTTCTGCCTGTCACATACGC |
| *TaSUT1*-Q-F | GATCGTCTCCCTCGCCCTCT |
| *TaSUT1*-Q-R | GGGATGTTGCCCTTGCCGAA |
| *TaSUT2*-Q-F | TACGGAGTCCTGCTCTGTCA |
| *TaSUT2*-Q-R | CTCGTCGCTTCCGAAAGTA |
| *TaSWEET11*-Q-F | GGACGCTCGCCTACTTCTT |
| *TaSWEET11*-Q-R | GCAGGAACTCCACGCTCTT |

**Table S3** F-values in three-way ANOVA analysis of plant dry weight and young leaf area in wheat seedlings at 10 days after low N treatment. Cultivar indicates the effects between the two cultivars; Priming indicates the effects between with and without drought priming; N-level indicates the effects between two N levels; * and ** indicate significant at 0.05 and 0.01 level, respectively. ns indicates no significant difference.

| Source of variation | Shoot dry weight | Root dry weight | Total dry weight | Young leaf area |
| --- | --- | --- | --- | --- |
| Cultivar | 12.924** | 12.46** | 15.428** | 31.833** |
| Priming | 21.257** | 30.731** | 27.95** | 17.346** |
| N-level | 198.979** | 126.805** | 85.532** | 45.942** |
| Cultivar × Priming | 0.617ns | 0.248ns | 0.626ns | 0.16ns |
| Cultivar × N-level | 0.435ns | 3.825ns | 1.085ns | 2.344ns |
| Priming × N-level | 3.888ns | 9.265** | 5.886* | 4.493ns |
| Cultivar × Priming × N-level | 2.806ns | 1.477ns | 2.975ns | 1.293ns |

**Table S4** F-values in three-way ANOVA analysis of Rubisco activity and maximum carboxylation rate (*V*cmax) in wheat seedlings at 10 days after low N treatment. Cultivar indicates the effects between the two cultivars; Priming indicates the effects between with and without drought priming; N-level indicates the effects between two N levels; * and ** indicate significant at 0.05 and 0.01 level, respectively. ns indicates no significant difference.

| Treatment time | Source of variation | Old leaf | | Young leaf | |
| --- | --- | --- | --- | --- | --- |
|  |  | Rubisco activity | *V*cmax | Rubisco activity | *V*cmax |
| 5DAT | Cultivar | 37.089** | 33.364** |  |  |
|  | Priming | 6.8* | 9.827** |  |  |
|  | N-level | 5.041* | 2.302ns |  |  |
|  | Cultivar × Priming | 0.146ns | 1.551ns |  |  |
|  | Cultivar × N-level | 6.88* | 3.042ns |  |  |
|  | Priming × N-level | 1.121ns | 0.188ns |  |  |
|  | Cultivar × Priming × N-level | 1.061ns | 5.573* |  |  |
| 10DAT | Cultivar | 27.674** | 30.108** | 45.57** | 44.815** |
|  | Priming | 9.37** | 12.675** | 21.87** | 29.554** |
|  | N-level | 95.414** | 139.44** | 62.459** | 80.229** |
|  | Cultivar × Priming | 0.095ns | 0.231ns | 0.104ns | 0.352ns |
|  | Cultivar × N-level | 0.805ns | 0.039ns | 1.358ns | 1.243ns |
|  | Priming × N-level | 2.465ns | 1.386ns | 7.079* | 9.58** |
|  | Cultivar × Priming × N-level | 0.022ns | 0.692ns | 0.288ns | 0.686ns |

**Table S5** F-values in three-way ANOVA analysis of Rubisco content, Rubisco activation state and Rubisco activase (RCA) activity in wheat seedlings at 10 days after low N treatment. Cultivar indicates the effects between the two cultivars; Priming indicates the effects between with and without drought priming; N-level indicates the effects between two N levels; * and ** indicate significant at 0.05 and 0.01 level, respectively. ns indicates no significant difference.

| Treatment time | Source of variation | Old leaf | | | Young leaf | | |
| --- | --- | --- | --- | --- | --- | --- | --- |
|  |  | Rubisco content | Rubisco activation state | RCA | Rubisco content | Rubisco activation state | RCA |
| 5DAT | Cultivar | 1.352ns | 23.488** | 21.354** |  |  |  |
|  | Priming | 8.349* | 1.405ns | 9.633** |  |  |  |
|  | N-level | 76.272 | 98.789 | 160.466 |  |  |  |
|  | Cultivar × Priming | 0.518ns | 0.447ns | 1.717ns |  |  |  |
|  | Cultivar × N-level | 1.741ns | 1.636ns | 0.977ns |  |  |  |
|  | Priming × N-level | 2.008ns | 4.511ns | 6.28* |  |  |  |
|  | Cultivar × Priming × N-level | 2.947ns | 0.231ns | 0.977ns |  |  |  |
| 10DAT | Cultivar | 14.414** | 26.535** | 38.007** | 8.961** | 39.284** | 19.731** |
|  | Priming | 8.637* | 4.752* | 19.947** | 16.965** | 6.331* | 20.195** |
|  | N-level | 910.644** | 59.828** | 121.929** | 171.533** | 141.126** | 251.904** |
|  | Cultivar × Priming | 0.118ns | 0.528ns | 0.007ns | 0.482ns | 0.43ns | 0.422ns |
|  | Cultivar × N-level | 1.064ns | 0.032ns | 4.685* | 0.008ns | 1.107ns | 0.567ns |
|  | Priming × N-level | 0.52ns | 5.946* | 14.091** | 4.501ns | 6.028* | 12.298** |
|  | Cultivar × Priming × N-level | 0.279ns | 0.138ns | 0.397ns | 0.475ns | 0.067ns | 0.648ns |

**Table S6** F-values in three-way ANOVA analysis of triose phosphate utilization rate (*V_TPU_*) and leaf inorganic phosphorus (Pi) concentration in wheat seedlings at 10 days after low N treatment. Cultivar indicates the effects between the two cultivars; Priming indicates the effects between with and without drought priming; N-level indicates the effects between two N levels; * and ** indicate significant at 0.05 and 0.01 level, respectively. ns indicates no significant difference.

| Treatment time | Source of variation | Old leaf | | Young leaf | |
| --- | --- | --- | --- | --- | --- |
|  |  | *V_TPU_* | Pi concentration | *V_TPU_* | Pi concentration |
| 5DAT | Cultivar | 50.682** | 16.877** |  |  |
|  | Priming | 10.438** | 8.903** |  |  |
|  | N-level | 14.613** | 32.092** |  |  |
|  | Cultivar × Priming | 0.114ns | 0.905ns |  |  |
|  | Cultivar × N-level | 3.241ns | 5.453* |  |  |
|  | Priming × N-level | 0.131ns | 3.299ns |  |  |
|  | Cultivar × Priming × N-level | 1.561ns | 2.036ns |  |  |
| 10DAT | Cultivar | 34.125** | 17.564** | 35.702** | 1.24ns |
|  | Priming | 41.266** | 6.254* | 19.622** | 11.931** |
|  | N-level | 112.788** | 92.281** | 5.241* | 73.753** |
|  | Cultivar × Priming | 0.078ns | 0.407ns | 0.033ns | 0.029ns |
|  | Cultivar × N-level | 1.5ns | 0.086ns | 0.207ns | 0.388ns |
|  | Priming × N-level | 8.829** | 9.53** | 4.201ns | 3.634ns |
|  | Cultivar × Priming × N-level | 0.784ns | 0.036ns | 2.262ns | 0.043ns |

**Table S7** F-values in three-way ANOVA analysis of sucrose phosphate synthase (SPS) activity and sucrose content in wheat seedlings at 10 days after low N treatment. Cultivar indicates the effects between the two cultivars; Priming indicates the effects between with and without drought priming; N-level indicates the effects between two N levels; * and ** indicate significant at 0.05 and 0.01 level, respectively. ns indicates no significant difference.

| Treatment time | Source of variation | Old leaf | | Young leaf | |
| --- | --- | --- | --- | --- | --- |
|  |  | SPS | sucrose content | SPS | sucrose content |
| 5DAT | Cultivar | 6.999* | 5.907* |  |  |
|  | Priming | 8.52* | 2.611ns |  |  |
|  | N-level | 15.168** | 25.636** |  |  |
|  | Cultivar × Priming | 0.415ns | 0.909ns |  |  |
|  | Cultivar × N-level | 3.612ns | 5.267* |  |  |
|  | Priming × N-level | 2.933ns | 5.956* |  |  |
|  | Cultivar × Priming × N-level | 1.047ns | 0.378ns |  |  |
| 10DAT | Cultivar | 4.116ns | 8.9** | 2.677ns | 7.259* |
|  | Priming | 12.665** | 6.718* | 20.124** | 15.958** |
|  | N-level | 8.001* | 110.054** | 0.003ns | 151.475** |
|  | Cultivar × Priming | 0.009ns | 0.268ns | 0.104ns | 0.536ns |
|  | Cultivar × N-level | 0.008ns | 3.491ns | 1.208ns | 2.854ns |
|  | Priming × N-level | 6.238* | 11.178** | 8.655* | 8.202* |
|  | Cultivar × Priming × N-level | 0.263ns | 0.218ns | 0.673ns | 1.254ns |

**Table S8** F-values in three-way ANOVA analysis of the relative expression of sugar transporter genes in wheat seedlings at 10 days after low N treatment. Cultivar indicates the effects between the two cultivars; Priming indicates the effects between with and without drought priming; N-level indicates the effects between two N levels; * and ** indicate significant at 0.05 and 0.01 level, respectively. ns indicates no significant difference.

| Treatment time | Source of variation | Old leaf | | | | Young leaf | | |
| --- | --- | --- | --- | --- | --- | --- | --- | --- |
|  |  | *TaSUT1* | *TaSUT2* | *TaSWEET11* | *TaSUT1* | | *TaSUT2* | *TaSWEET11* |
| 5DAT | Cultivar | 5.455* | 6.224* | 17.667** |  | |  |  |
|  | Priming | 41.812** | 46.621** | 81.788** |  | |  |  |
|  | N-level | 251.433** | 202.8** | 262.842** |  | |  |  |
|  | Cultivar × Priming | 0.189ns | 0ns | 0.164ns |  | |  |  |
|  | Cultivar × N-level | 3.953ns | 7.603* | 10.625** |  | |  |  |
|  | Priming × N-level | 10.391** | 24.897** | 40.449** |  | |  |  |
|  | Cultivar × Priming × N-level | 0.611ns | 0.082ns | 1.817ns |  | |  |  |
| 10DAT | Cultivar | 12.771** | 5.544* | 13.405** | 6.954* | | 8.126* | 7.797* |
|  | Priming | 38.875** | 29.965** | 15.602** | 42.732** | | 34.739** | 34.708** |
|  | N-level | 18.29** | 0.933ns | 22.756** | 153.587** | | 41.459** | 111.087** |
|  | Cultivar × Priming | 0.261ns | 1.053ns | 0.133ns | 0.044ns | | 0.29ns | 1.206ns |
|  | Cultivar × N-level | 6.688* | 1.818ns | 10.969** | 2.835ns | | 0.134ns | 0.995ns |
|  | Priming × N-level | 22.201** | 21.8** | 8.957** | 18.244** | | 8.744** | 12.057** |
|  | Cultivar × Priming × N-level | 2.244ns | 3.97ns | 0.002ns | 1.297ns | | 3.688ns | 0.517ns |
